# Characteristics of Self-Healable Copolymers of Styrene and Eugenol Terminated Polyurethane Prepolymer

**DOI:** 10.3390/polym11101674

**Published:** 2019-10-14

**Authors:** Jing-Yu Liang, Se-Ra Shin, Soo-Hyoung Lee, Dai-Soo Lee

**Affiliations:** 1 Department of Semiconductor and Chemical Engineering, Chonbuk National University, 567 Baekje-daero, Deokjini-gu, Jeonju 54896, Korea; liangjy@naver.com (J.-Y.L.); srshin89@jbnu.ac.kr (S.-R.S.); 2 Research Institute of Future Energy, Chonbuk National University, 567 Baekje-daero, Deokjini-gu, Jeonju 54896, Korea

**Keywords:** eugenol, styrene, self-healing, copolymers, polyurethane prepolymer

## Abstract

With limited biomass that can be currently utilized as a renewable resource, it is important to develop a method to convert biomass into materials that can replace fossil fuel product. In this paper, eugenol, a bio-based allyl chain-substituted guaiacol, was used to synthesize self-healable copolymers. Eugenol terminated polyurethane prepolymer (ETPU) was synthesized from eugenol and polyurethane prepolymers terminated with isocyanate groups. ETPU contained two allyl groups. Self-healing copolymer networks were obtained by copolymerization of ETPU and styrene monomer via free radical polymerization. Effects of ETPU content on the properties of copolymers were then studied. These copolymers containing ETPU exhibited good thermal stability and mechanical properties. These copolymers showed higher tensile strength and elongation at break than PS. Their maximum tensile strength reached 19 MPa. In addition, these copolymers showed self-healing property at elevated temperature due to the reversible nature of urethane units in ETPU.

## 1. Introduction

Due to continuously increasing price of petroleum resources and increase in environmental awareness, biomass derived from renewable resources has attracted a great deal of attention. For the practical applications of the biomass for polymers, efforts to find advantages in cost, performance, and LCA are necessary. Several biomasses have been proposed to prepare new bio-based materials, including vegetable oils [1], fatty acids [2], lignin [3], vanillin [4], succinic acid [5], and eugenol [6]. 

Self-healing is a process of healing microcracks to extend the safety and working life of polymeric materials. So far, major findings in this field can be divided into two categories: extrinsic and intrinsic self-healing [7]. The extrinsic healing system includes microcapsules or hollow fibers releasing healing agents after crack formation. The intrinsic healing is based on reversible covalent or non-covalent supramolecular interactions. Non-covalent methods are based on hydrogen bonding [8], ionic interactions, or metal-ligand interaction [9]. However, non-covalent bonding-based polymers showed weak mechanical properties [10]. Thus, reversible dynamic covalent bonds such as disulfide bonds [11], nitroxide bonds [12], acylhydrazone bonds [13], Diels-Alder (DA) reaction products [14], urethane bonds [15], and reversible ester bonds [16] have been receiving increasing attention. These reversible covalent bonds can especially contribute to the healing ability of polymers by various stimuli. 

Polyurethanes (PUs) are among the most important polymeric materials. Having a wide range of density, hardness, and stiffness, they have been widely used as adhesives, foams, and coatings. The PUs prepared from three main components, long chain diols (polyols), diisocyanates (aromatic or aliphatic types) and short chain diols (chain extenders) are segmented copolymers of hard and soft segment domains. Recently, many studies have focused on the self-healing properties of PUs [17,18,19]. Self-healing PUs repair damage by the release of healing agents or temporary increased mobility. There are many different types of polyols, chain extenders, and isocyanates, so the choices of self-healing mechanism for PUs are diverse. The success of the healing capability depends on the characteristics of the dynamic nature of the structures. For the reversible covalent bonds, DA reaction product is probably widely known. Feng et al. studied a new thermoplastic polyurethane (TPU) with excellent self-healing performance due to the incorporated DA reaction products, and the cracks in polyurethane films could be repaired by dual actions of thermo-reversible DA reaction and thermal movement of molecular chains [20]. Lin and co-workers synthesized crosslinker with maleimide groups and used in the reactions with the pendant furan groups of PU to prepare the self-healing electrically conductive composites based on DA reaction [21]. Polymers with disulfide groups can undergo self-healing even at room temperature. Kim and co-workers developed a transparent and quickly self-healing TPU at room temperature due to the aromatic disulfide structure [22]. Yangisawa reported complete healing of PU elastomer at 36 °C via hydrogen bonding of thiourea [23]. Urethane bonds in traditional PUs are made from hydroxyl groups of polyols, and various polyisocyanates. These bonds are reversible at elevated temperature [24,25]. When polyisocyanate reacts with aliphatic hydroxyls, PUs that contains thermodynamically stable bonds can be formed. Temperatures for the retro reactions of urethanes are important factors that allow self-healing of PUs. It should be as low as possible. The main dissociation pathway depends on the equilibrium between urethane bond and the starting functional group. When temperature increases, reverse reaction occurs. Electronic effect of substituents also influences the kinetics and equilibrium of thermally reversible reactions of urethane bonds. Aromatic hydroxyl group-based PUs favor the reverse reaction. According to reactivity classification, thermal dissociation temperatures are in the following order: alkyl-NCO/alkyl-OH (250 °C) > aryl-NCO/alkyl-OH (200 °C) > alkyl-NCO/aryl-OH (180 °C) > aryl-NCO/aryl-OH (120 °C) [26]. Phenolic hydroxyl can react with isocyanate to form phenolic urethane that can undergo dissociations into phenol and isocyanate due to the dynamic natures.

Eugenol (4-allyl-2-methoxyphenol) is a natural allyl chain-substituted guaiacol with allyl group extracted from lignocellulosic biomass. It can also be obtained from pyrolysis [27,28] or depolymerization [29] of lignin. It is a relatively cheap bio-based compound with multi-reactive groups. It has great potential to be widely used in polymers due to its unique structure, abundant availability, and low cost. Eugenol is also used in perfumes, antioxidants, drugs, and foods [30]. In recent years, eugenol has attracted major interest in the development of bio-based polymers. Considering its potential abundant production and unique structure which includes methoxy-substituted phenolic ring and allyl group, it has been regarded as an ideal candidate to synthesize petroleum-based compounds such as thermosetting resins [31], benzoxazines resins [32], and epoxy resins [33]. 

Polystyrene (PS) is clear, hard, and brittle, but slow to biodegrade [34]. Therefore, it has been a focus of controversy among environmentalists. Styrene can easily copolymerize with different monomers such as acrylonitrile, acrylic monomers, butadiene, maleic anhydride, unsaturated polyesters, and others to create polymeric materials with unique properties suitable for many industrial applications [35,36,37]. In order to improve the processing, performance, and elasticity of plastic materials, polar and non-polar additives such as plasticizing modifiers can be added, resulting in materials with lower moduli, stiffness, and glass transition temperature. Meanwhile, elongation ability and polymer chain flexibility of materials are significantly increased [38,39]. In recent years, the utilization of eco-friendly modifiers such as epoxidized vegetable oils, biodiesel oils, hydrogenated Castrol oil, citrate esters, and poly (ethylene glycol) of low molecular weight has been investigated [40,41,42].

In this study, thermal and mechanical properties of PS modified with eugenol derivatives were investigated to impart toughness and self-healing capability to PS. Especially, eugenol terminated polyurethane prepolymers (ETPU) containing two vinyl groups at both ends were synthesized. Through free-radical polymerization process, copolymers of ETPU and styrene monomer were prepared. The influence of ETPU on copolymers was studied. Thermal properties, mechanical properties, and self-healing properties of these copolymers were investigated. Copolymers with aromatic urethane bonding structures showed considerable reverse reactions and implement the self-healing properties. It is postulated that ETPU can give vinyl polymers self-healing properties by copolymerizations due to the dynamic nature of urethane units based on eugenol.

## 2. Experimentals 

### 2.1. Materials

Poly (tetramethylene ether glycol) (PTMEG1000) (Number average molecular weight: 1000g/mol, 99%), eugenol (99%), styrene monomer (SM, 99%), dibutyltin dilaurate (DBTDL, 95%), and tert-butyl peroxybenzoate (TBPB, 98%), purchased from Sigma-Aldrich Chemical (Yongin City, Korea). And 4,4’-Methylene bis(phenyl isocyanate) (MDI, 99%) was obtained from BASF (BASF Korea, Yeosu, Korea). Dimethylformamide (DMF, 99%) was supplied by Samchun Pure Chemical (Pyeongtaek-si, Korea).

### 2.2. Synthesis of Eugenol-terminated Polyurethane (ETPU)

**Scheme 1:**
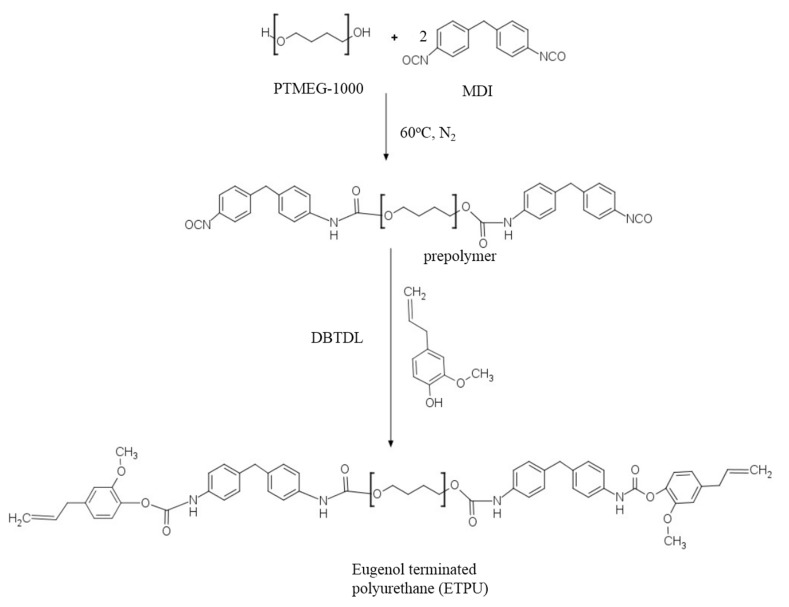
Synthesis and structure of eugenol-terminated polyurethane prepolymer.

In a 500 mL round-bottomed flask, PTMEG-1000 (200 g, 0.2 mol) and MDI (100 g, 0.4 mol) were mixed and stirred at 60 °C under N_2_ gas environment. After the mixture became homogeneous, the temperature was kept at 60 °C and reaction proceeded without any solvent or catalyst. With continued stirring until NCO% reached the theoretical value based on the stoichiometry, eugenol (65.6 g, 0.4mol) and DBTDL (0.73 g, 0.2wt.% based on the total weight of mixture) were added into PU prepolymers. After mixing and stirring at 70 °C for about 5 h, the isocyanate group completely reacted (NCO% below 0.1%). NCO% was determined following ASTM D1638-74. Preparation route and ETPU chemical structure are shown in Scheme 1.

### 2.3. Preparation of Copolymers

**Scheme 2:**
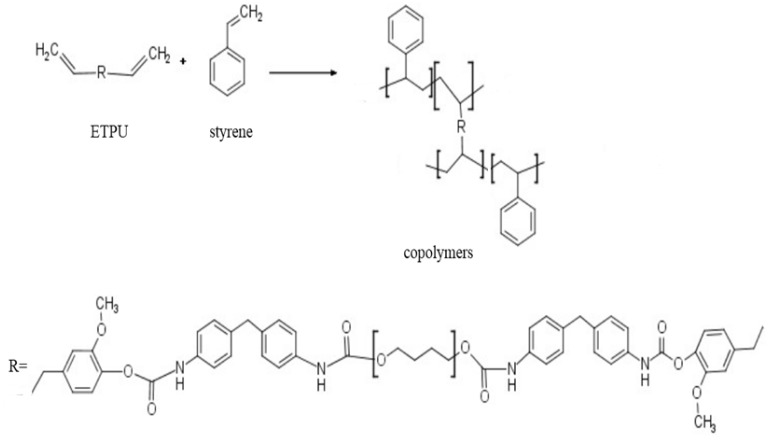
Synthesis and structures of crosslinked copolymers.

Detailed formulations of copolymers are shown in Table 1. In this experiment, TBPB (1 wt.% based on total weight of monomer mixtures) was used as an initiator. Different contents of styrene monomers were mixed with ETPU. These mixtures of SM and ETPU were stirred at 100 °C for several minutes to obtain a homogeneous solution. After pouring into silicon film molds, they were cured at 110 °C in a convection oven for 1 day. Chemical structures of copolymers are shown in Scheme 2.

### 2.4. Characterizations

To confirm ETPU synthesis and identify the isocyanate group released by the reversible reaction of urethane at elevated temperatures (100–200 °C), the copolymers were analyzed by FT-IR spectroscopy (FT-IR-302, Jasco, Tokyo, Japan). Differential scanning calorimetry was performed using a Q20 DSC from TA instrument (New Castle, DE, USA) with heating rate of 10 min^−1^ from −80 °C to 200 °C in nitrogen gas environment. Thermogravimetric analysis (TGA) was performed using Q500 TGA from TA instrument (New Castle, DE, USA) under the following condition: heating rate of 20 °C / min, temperature range of 20–800 °C, sample weight range of 10-20 mg, and nitrogen flow at 60 mL/min. ^1^H-nuclear magnetic resonance (NMR) was performed using a 600 MHz JNM-ECA600 spectrometer (600 MHz, JNMECA600, JEOL, Tokyo, Japan) with CDCl_3_ as solvent. Tensile properties and self-healing efficiency were measured using a universal testing machine (UTM, LR5K Plus from LLOYD, West Sussex, UK). Measurements were carried out at 25 °C with cross-head speed of 500 mm min^−1^ following the ASTM-D638 method. Dynamic mechanical analysis (DMA) was conducted using a Q800 from TA instrument (New Castle, DE, USA). The dimension of sample size was 16 mm × 5 mm × 1.5 mm and the heating rate was 5 °C min^−1^ from −100 °C to 200 °C and the frequency was set at 1 Hz. Atomic force microscope (AFM, Multimode-8, Bruker, Billerica, USA) was employed to observe morphologies of the copolymers. Self-healing abilities of films were observed with an optical microscope with fluorescence filters coupled with a digital camera. Fresh cuts were made using a sharp blade on surfaces of films. They were placed in an oven preset at 150 °C for 24 h and then cooled down to room temperature. The healing efficiency was obtained from cut & heal tests in tensile property measurements with the following equation:

Healing efficiency = (σ_healing_/σ_original_) × 100% (1)

where σ_healing_ and σ_original_ are tensile strength of healed specimen and original specimen respectively. The self-healing properties of the samples also were investigated using an AIS2100 scanning electron microscope (SEM; Seron Technologies Inc., Gyeonggi-do, Korea). Copolymer specimens of 5 mm × 5 mm were fixed with a carbon tape on the plates for scratch-healing test. A thin scratch was made on the surface of copolymer specimens using a razor blade. The copolymer specimens was coated with gold using an ion coater (HC-21, Hoyeontech, Seongnam, Korea). SEM images were taken after the heat treatment of the scratched specimens of the copolymers in a vacuum oven at 150 °С for 12 h. The swelling ratios of the copolymer specimens in DMF were measured. It was found that the samples reached the equilibrium swelling after 24 h at room temperature. Excessive solvent on the surface was removed with filter paper and the fully swollen samples were weighed. The swelling ratios were calculated employing the following equation:

Swelling ratio(%)=((W_s_−W_d_)/W_d_) × 100 (2)

where W_s_ is the weight of the sample in the swollen state and W_d_ that of the dry state of the sample. 

## 3. Results and Discussion

### 3.1. Synthesis and Characterization of ETPU 

The synthesis pathway of ETPU is shown in Scheme 1. NCO-terminated prepolymers were synthesized from the reaction between MDI and PTMEG1000. When NCO% reached the theoretical value, the prepared NCO-terminated prepolymer was capped with eugenol to obtain ETPU.

**Figure 1:**
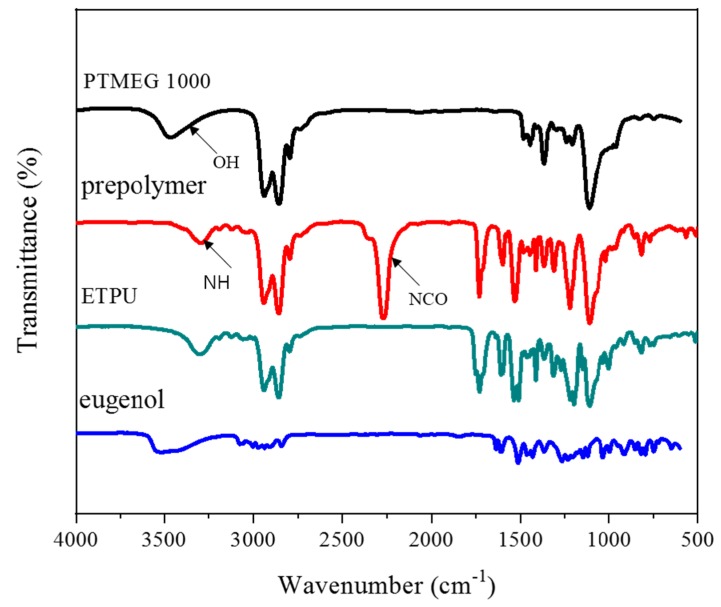
FT-IR spectra of Poly (tetramethylene ether glycol) (PTMEG), prepolymer, eugenol, and eugenol terminated polyurethane prepolymer (ETPU).

**Figure 2:**
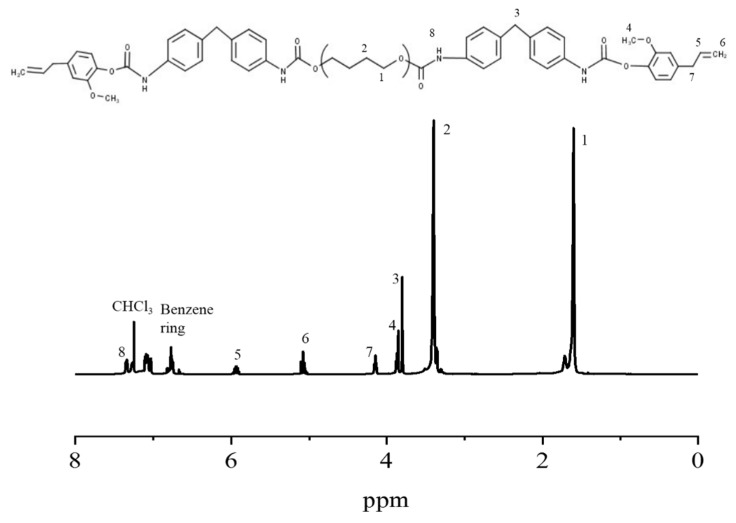
^1^H-NMR spectrum of ETPU.

FT-IR spectra of PTMEG1000, prepolymer, eugenol, and ETPU are shown in Figure 1. After PTMEG1000 reacted with MDI, the prepolymer showed strong band at 2270 cm^−1^ due to the presence of free N=C=O groups of prepolymer. Peaks at 1739 cm^−1^ and 1600 cm^−1^ corresponded to stretching vibration of C=O and N-H bonds in urethane groups, respectively. Compared with prepolymer, the –OH peak observed in PTMEG1000 at 3462 cm^−1^ disappeared while N-H stretching vibration at around 3298 cm^−1^ was observed in FT-IR spectra of prepolymer due to successful synthesis of prepolymer. After the prepolymer reacted with eugenol, the –N=C=O absorption peak at 2270 cm^−1^ completely disappeared. The peak at 3510 cm^−1^ corresponding to -OH of eugenol also disappeared due to the completion of prepolymer capping process with eugenol. ^1^H-NMR spectra of ETPU are shown in Figure 2. Signals observed at 7.2 ppm were assigned to urethane group. Peaks at 5.9 ppm and 5.1 ppm corresponded to carbon double bond (–CH=CH_2_) present in eugenol. Peaks at 1.4 ppm and 3.2 ppm were assigned to CH_2_ bond from PTMEG. FT-IR analysis results also revealed that the reaction between prepolymer and eugenol was completed. It was postulated that ETPU was successfully synthesized by Scheme 1.

**Figure 3:**
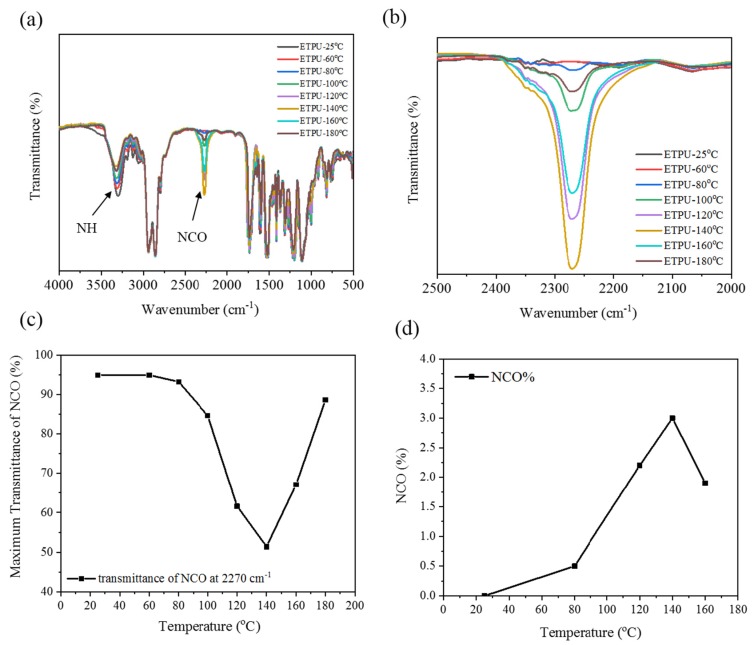
Variable-temperature FT-IR spectra of ETPU with reversible urethane bonds: (**a**) FT-IR spectra for the copolymers at 4000~500 cm^−^^1^; (**b**)FT-IR spectra at 2000~2500 cm^−^^1^; (**c**) Transmittance change at 2270 cm^−1^ at elevated temperatures; (**d**) Change of NCO% at elevated temperatures.

Variable-temperature FT-IR displaying changes in covalent bonds was used to track the dynamic process of disassociation and reconnection of reversible urethane bonds in ETPU. FT-IR spectra of ETPU scanned from 25 °C to 180 °C are shown in Figure 3. As shown in Figure 3b, the –NCO peak at 2270 cm^−1^ was not observed at 25 °C. However, at temperature above 80 °C, it appeared since the urethane group started reverse reactions at this temperature. When the temperature was increased to 140°C, the intensity of NCO peak became stronger while that of the NH peak at 3298 cm^−1^ was decreased, indicating that there was a reversible nature of urethane bonds formed from phenolic hydroxyl and NCO groups [25]. However, when the temperature was further increased to above 150 °C, the -NCO peak started to weaken. The decrease of peak intensity due to -NCO groups can be attributable to the reaction of isocyanate to form allophanate, which is faster than the retro reactions of urethane bonds into hydroxyl and isocyanate groups.

In order to confirm the reversible features of urethane bonds in ETPU additionally, NCO contents (%) of ETPU after heat treatments for 30 min at elevated temperatures were determined by titration and given in Figure 3d. Change of NCO contents showed the same trends as the change of -NCO peak intensity in Figure 3c. It is suggested that urethane bonds of ETPU are reversible at elevated temperatures investigated as Lee reported [24].

### 3.2. Thermal Stability of Copolymers

**Figure 4:**
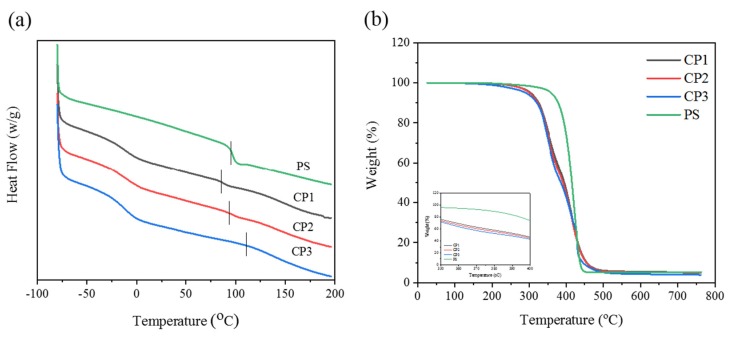
Thermal properties of copolymers: (**a**) DSC thermograms; (**b**) TGA curves.

The curing behavior of copolymers was measured by DSC. Figure 4a shows DSC curves after curing reaction. As shown in Figure 4a, no more exothermic peaks were observed, confirming that the polymerization was completed. Glass transition temperature (T_g_) values of copolymers were obtained by DSC analysis. DSC curves showed that copolymers had two glass-transition temperatures at about −20 °C and 90 °C possibly due to soft domain of ETPU and polystyrene phase, respectively. PS only showed one T_g_ at 98 °C. CP1, CP2, and CP3 copolymers showed a soft segment domain T_g_ (T_gs_) at −12 °C and the hard segment T_g_ (T_gh_) were increased with the increase of ETPU content. The soft T_gs_ was due to the presence of polyurethane chains while the hard T_gh_ was due to the polystyrene matrix in copolymers. Although the flexible ETPU was introduced to the styrene matrix, the hard T_gh_ increased with increasing ETPU content. This is because when the ETPU content is increased, the crosslinking density of copolymers is also increased. The increased crosslinking density might have reduced the molecular mobility of copolymers, thus increasing T_gh_. 

TGA was performed to investigate thermal stability of copolymers. Figure 4b shows TGA curves under nitrogen atmosphere. Detailed data are given in Table 2. Pure PS decomposed in one main stage that was visible from 340 °C to almost 430 °C. It showed the highest thermal stability. The addition of ETPU to PS resulted in displacement of the initial decomposition temperature of obtained materials toward lower temperatures. Meanwhile, an increase in the number of degradation stages was observed together with the presence of ETPU. These copolymers decomposed in two main stages. The first degradation stage appeared at lower temperatures due to decomposition of urethane moieties. Thermal degradation temperature for 5% weight loss (T_d 5%_) decreased with increasing content of ETPU. This indicates that, with increasing ETPU content, thermal stability is lowered. Furthermore, the residue content at 700 °C was decreased with increasing ETPU content. 

### 3.3. Mechanical Properties of the Copolymers

**Figure 5:**
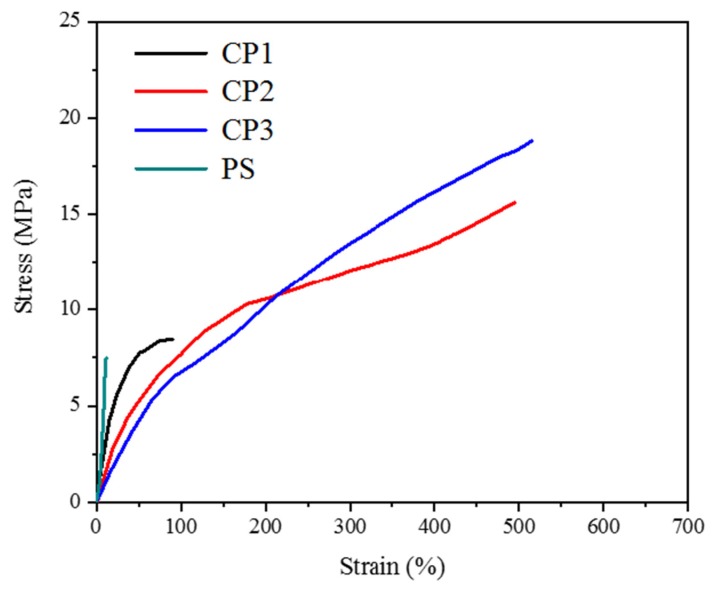
Stress-strain curves of copolymers with different ETPU contents.

Stress-strain curves for the different copolymers in tensile tests are shown in Figure 5 and tensile properties are summarized in Table 2. It is observed that Young’s moduli decrease while tensile strengths and elongations at break increase as the content of ETPU in the copolymers are increased. It is speculated that the incorporation of flexible ETPU segments in the copolymers resulted in the decrease of the modulus. It is of interest to note that the ETPU segments of the copolymers imparted toughening to the copolymers, leading to the improvement of tensile strength and elongation at break. CP1 showed slightly enhanced tensile strength with slightly increased elongation at break, because the ETPU not only act as cross-linker for PS, but also increased the flexibility of copolymers. For the CP2 and CP3, with the ETPU content increased, the crosslinking density and soft segment content increased, resulting in the large improvement of tensile strengths and elongations at break. It is inferred that the increase of ETPU content in the copolymers led to an improvement of chain flexibility, resulting in the increase of the tensile strength and elongation at break efficiently.

### 3.4. Dynamic Mechanical Properties 

**Figure 6:**
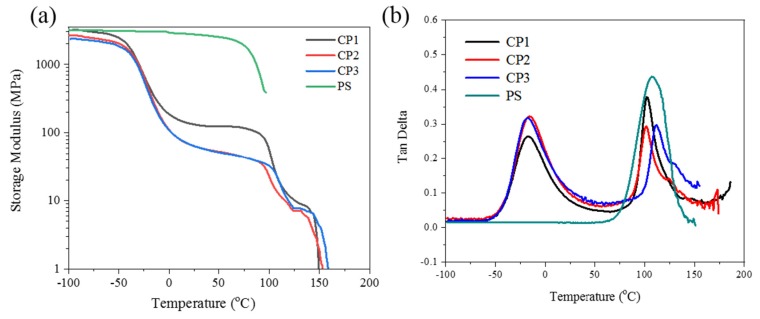
Dynamic mechanical analysis (DMA) curves for copolymers: (**a**) Storage modulus of copolymers; (**b**) Tan delta of copolymers.

**Figure 7:**
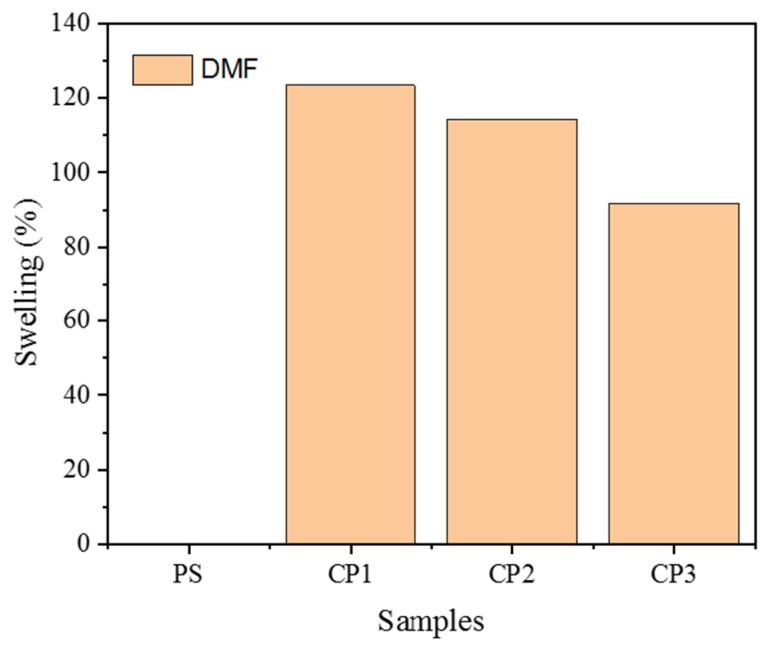
Swelling ratio of the copolymers in DMF at room temperature after 24 hrs.

Dynamic mechanical thermal measurements were used to examine viscoelastic properties of the copolymers. Figure 6 shows temperature dependence of storage modulus and loss factor of the copolymers. PS showed one T_g_ at 105 °C. It had the highest storage modulus in Figure 6. Although cross-linked structure was introduced by addition of ETPU to PS, the storage modulus of copolymer was gradually decreased with increasing content of ETPU. This is because of the decrease in rigidity of copolymer caused by ETPU. T_g_ values of copolymers in this study were determined by maximum peak temperatures of tan delta values as shown in Figure 6b. Copolymers exhibited two T_g_s due to co-existence of PU segments connected with polystyrene chains. This result coincides with DSC results. T_g_ values of copolymers networks were affected by crosslinking and the content of soft segment. Furthermore, these two peaks showed damping due to transitions of soft PU domain and hard polystyrene matrix. All copolymers were found to have similar T_gs_ due to ETPU chains. On the other hand, T_gh_ of hard polystyrene chains increased with increasing ETPU content due to the increased crosslinking density of copolymers. Network properties of the crosslinked polymers can be investigated by swelling tests in organic solvents. In order to study the crosslinking of the copolymers, swelling properties of the copolymers in DMF were measured at room temperature. Figure 7 showed the swelling ratios of the copolymers after immersing the specimens in DMF for 24 hrs. It was observed that swelling ratios of the copolymers decreased with increase of the ETPU content, implying the crosslinking density increased with increase of the ETPU content. These DMA data and swelling properties of the copolymers demonstrate that ETPU can significantly influence chemical properties as well as the mechanical properties of copolymers by introducing network structures. Table 2 gives summary of tensile properties and thermal properties of copolymers.

### 3.5. Atomic Force Microscopy of Copolymers

**Figure 8:**
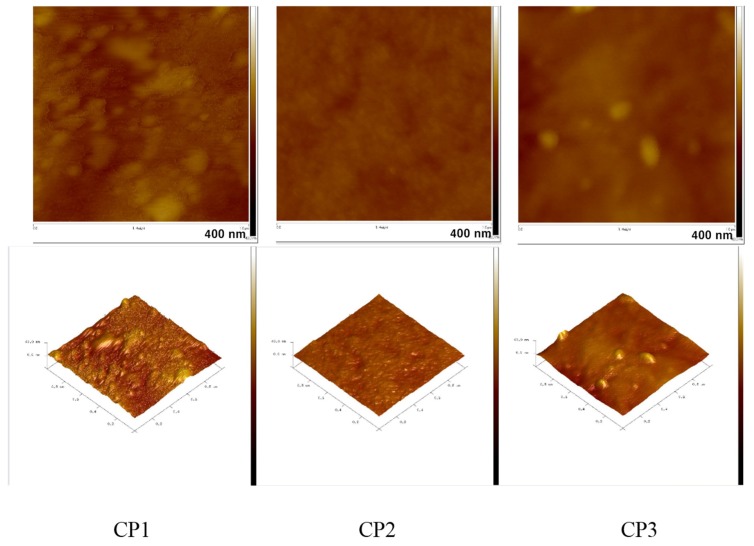
AFM images of copolymers.

To understand the influence of content of soft segment (ETPU) on the formation of surface topography and heterogeneity of copolymers, AFM analysis was performed to investigate the morphology of copolymers. As shown in Figure 8, 3D phase/height contrast images of copolymers surface were observed. It is known that bright regions represent the hard phase (hard domains) while darker regions represent the soft phase in a segmented polyurethane. The phase indicated the distribution of the copolymers with different hardness. Images showed phase separation due to hard phase of polystyrene and soft phase of ETPU. With increasing ETPU content, soft domains showed increases. In comparison with different contents of ETPU, roughness values of R_q_ for CP1, CP2, and CP3 copolymers were 2.77 nm, 3.03 nm, and 10.1 nm, respectively. The roughness increased with increasing content of ETPU. When the content of ETPU was lower (CP1), most phases were hard domains showing lower roughness. With the increase of ETPU content (CP2 and CP3), the number of soft domains increases, leading to increase of roughness. Thus, sharp interface between both phases was observed, indicating increased heterogeneity. These synthesized copolymers were characterized by two-phase morphology consisting of hard segment-rich phase and soft segment-rich phase. It is evident that these synthesized copolymers can be distinguished by the heterogeneous character. As shown in Figure 8, styrene and ETPU contents have significantly effects on the structure of copolymer. These results were consistent with DSC data.

### 3.6. Self-healing Properties 

**Figure 9:**
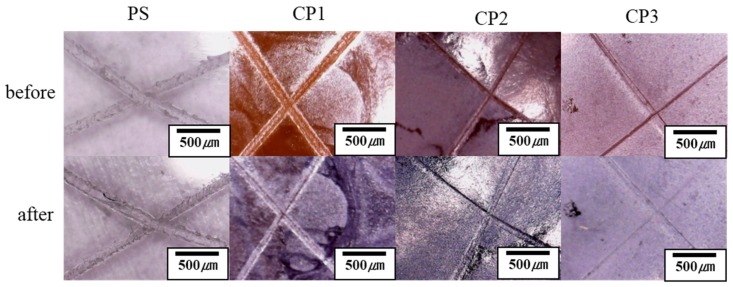
Microscope images of copolymers at 150 °C before and after thermal treatment.

**Figure 10:**
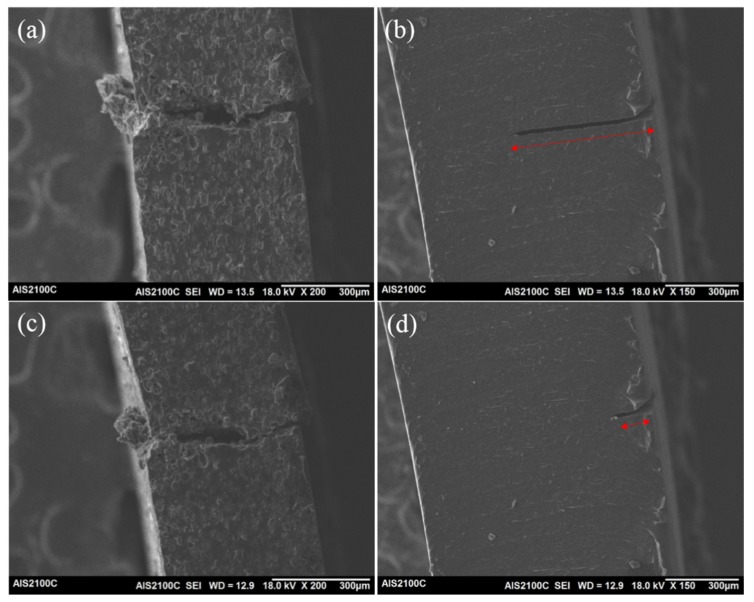
SEM images of copolymers before and after heating treatment: (**a**) CP1 before healing; (**b**) CP3 before heated; (**c**) CP1 after healing at 150 °C for 12hr; (**d**) CP3 after healing at 150 °C for 12hr.

To prove the healing ability, self-healing tests were carried out first by scratching the surface of copolymer. The damage was then thermally treated at 150 °C for 24 h. Thermal healing of the copolymers were recorded by digital camera. Results are shown in Figure 9. Even after thermal treatment, PS or CP1 did not show healing properties, recovery of scratches. The scratch was seen clearly on the surface due to the absence or very low concentration of urethane bond which showed reversibility with heating (Figure 3). On the other hand, the scratch of CP2 and CP3 showed healing properties after thermal treatment, although the scratch was not completely recovered. At this temperature, the reversible urethane bond disassociated and reconnected, leading to urethane bond reformation, thus showing healing properties on the surface. Based on these results, CP2 and CP3 were used to test the healing efficiency by measuring the mechanical properties. To investigate the self-healing properties further, a scratch healing test was conducted additionally. Here, specimens of CP1 and CP3 were scratched using a razor blade, and the cross-section of the copolymer specimens were observed employing SEM before and after the thermal healing. As shown in Figure 10, we could confirm that the depth of scratch in CP3 decreased largely after the healing while that of CP1 hardly changed. 

**Figure 11:**
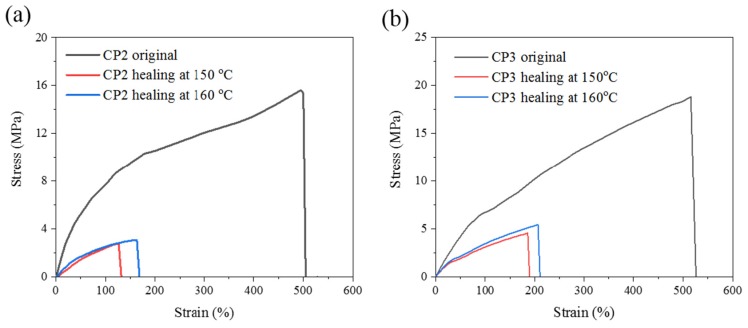
Stress-strain curves of the copolymers after healing at 150 °C and 160 °C for 24hr: (**a**) CP2; (**b**) CP3.

Mechanical properties are critical for self-healing materials. In this experiment, the sample was cut into half and connected with the original section for self-healing for 24 h at 150 °C and 160 °C. In order to investigate self-healing properties, strain–stress curves were acquired for all samples. Figure 11 presents results of self-healing test in mechanical properties of copolymers with different ETPU contents. Data of tensile strength (σ) and elongation at break (ε) before and after the healing are summarized in Table 3. CP2 and CP3 showed thermally healing properties after they were treated at elevated temperature (Figure 11). However, CP1 did not show healing properties due to their low contents of reversible urethane groups. Although CP2 and CP3 were not fully restored to their original mechanical properties, it was clear that when the content of ETPU was increased, the efficiency of self-healing was also increased due to reversible urethane unit in the ETPU. Increasing the temperature from 150 °C to 160 °C increased the reversible rate and the self-healing efficiency. These results suggest that the content of urethane bonds could affect self-healing properties and that styrene content can influence mechanical properties. Thus, copolymers having higher content of urethane bonds had better self-healing efficiency. 

**Figure 12:**
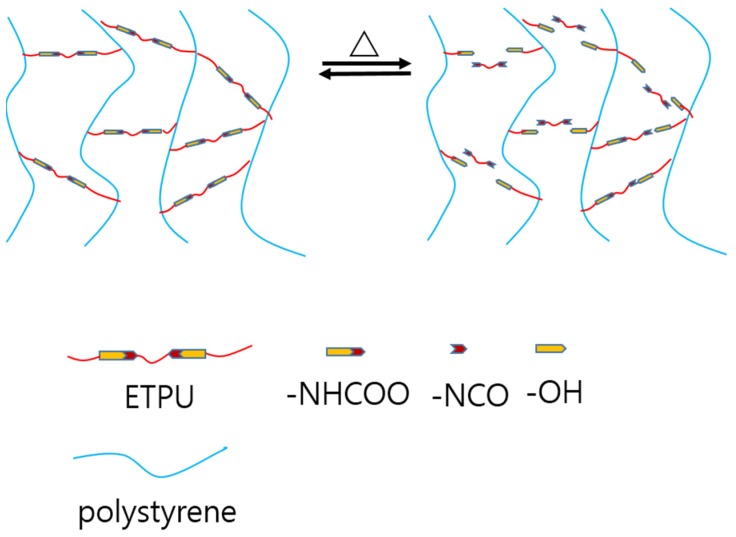
Self-healing mechanism of copolymers.

The self-healing mechanism of polyurethane can be explained as shown in Figure 12. The healing occurs at soft segment PU domain. Phenolic hydroxyl groups can react with isocyanate and phenolic urethane undergo retro reaction into phenol and isocyanate due to the dynamic natures which had been applied for the temporary masking of isocyanates with phenolic blocking agents through reversible reaction. After polymerization between styrene monomers and ETPU, the urethane bonds from the reaction of isocyanate and phenol were incorporated into the copolymers. It is stable at room temperature. However, reversible reactions occurs at elevated temperature. When the temperature cools down, the stable urethane bond forms again [24,25].

## 4. Conclusions

In this paper, ETPU, a eugenol-derived bio-based polyurethane prepolymer that contained two polymerizable groups, was successfully prepared and copolymerized with styrene monomers by free radical polymerization. ETPU showed NCO peaks at elevated temperatures due to reversible urethane units formed from phenolic hydroxyl group of eugenol and MDI. Results of this study confirmed that the addition of ETPU to PS resulted in the softer and more flexible materials. As a consequence, such copolymers had lower values of glass transition temperature, storage modulus, heat resistivity, and higher values of stress and strain at break compared to PS. Results of this study demonstrate that ETPU can be utilized as an internal, environmentally friendly modifier for vinyl polymers such as PS. The copolymer containing less ETPU showed little self-healing properties. With increasing content of ETPU, tensile strength and elongation at break of copolymers were increased due to more crosslinking by soft PU segment. It was observed that the thermal stability of the copolymer was also improved with increasing ETPU content in the copolymer. Two damping peaks were observed in DMA attributable to transitions of soft PU domain and hard polystyrene matrix, respectively. These copolymers also showed self-healing properties at elevated temperatures due to reversible features of urethane units in ETPU. Their self-healing efficiency also increased with increasing ETPU content in copolymers. 

## Figures and Tables

**Table 1:** Feed composition of copolymers.

Sample Codes	Monomer 1 Styrene (mol)	Monomer 2 ETPU (mol)	ETPU Content	
			mol%	wt.%
PS	10	0	0	0
CP1	9.8	0.2	2	26.4
CP2	9.6	0.4	4	42.3
CP3	9.1	0.9	9	63.5

**Table 2:** Summary of tensile properties, DMA, and TGA data of copolymers.

Sample codes	Tensile Strength (MPa)	Elongation at Break (%)	Storage Modulus at 20 °C (MPa)	T_g_ (°C)				T _d 5%_ (°C)	Residues at 700 °C (%)
				DMA		DSC			
				T_gs_	T_gh_	T_gs_	T_gh_		
PS	7.1	10	2480	0	105	0	98	358	5.3
CP1	8.4	80	138	−16	101	−11	90	303	5.2
CP2	15.4	504	72	−16	100	−12	101	300	4.9
CP3	18.8	600	71	−16	114	−12	109	290	3.9

**Table 3:** Self-healing properties of copolymers at different temperatures.

Sample	Original		After Healing				Healing Efficiency (%)	
			150 ℃		160 ℃		150 ℃	160 ℃
	σ (MPa)	ε (%)	σ (MPa)	ε (%)	σ (MPa)	ε (%)		
PS	7.1	10	-	-	-	-	-	-
CP1	8.4	80	-	-	-	-	-	-
CP2	15.6	504	2.78	126	3.08	163	17.8	19.7
CP3	18.8	600	4.6	190	5.5	206	24.5	29.2
